# Perspectives of non‐physician partners on barriers and facilitators to AYA cancer care in Latin America

**DOI:** 10.1002/cam4.70198

**Published:** 2024-10-02

**Authors:** Samantha L. Wong, Elysia Alvarez, Emily E. Johnston, Crystal Romero, Nuria Rossell, Ligia Rios, Wendy Gómez García, Federico Antillon‐Klussmann, Ligia Fu, Soad Fuentes‐Alabi, Karina Quintero Delgado, Daniel Ortiz Morales, Carolina Rodriguez‐Loza, Salvador A. Lopez, Melissa Gosdin, Marcio Malogolowkin, Paola Friedrich

**Affiliations:** ^1^ Division of Pediatric Hematology and Oncology Davis School of Medicine, University of California Sacramento California USA; ^2^ Heersink School of Medicine University of Alabama at Birmingham Birmingham Alabama USA; ^3^ Institute for Cancer Outcomes and Survivorship, Heersink School of Medicine University of Alabama at Birmingham Birmingham Alabama USA; ^4^ Independent Medical Anthropology Researcher San Salvador El Salvador; ^5^ Unidad de Oncología Pediátrica y del Adolescente Hospital Nacional Edgardo Rebagliati Martins Lima Peru; ^6^ Dr. Robert Reid Cabral Children's Hospital Santo Domingo Dominican Republic; ^7^ National Cancer Institute INCART Santo Domingo Dominican Republic; ^8^ Unidad Nacional de Oncología Pediátrica Guatemala City Guatemala; ^9^ School of Medicine Francisco Marroquín University Guatemala City Guatemala; ^10^ Hospital Escuela Tegucigalpa Honduras; ^11^ National Program for Childhood Cancer Ayudame a Vivir Foundation/National Children's Hospital Benjamin Bloom San Salvador El Salvador; ^12^ Hospital del Niño Dr. José Renán Esquivel Oncología Panama City Panama; ^13^ Hospital General de México Mexico City Mexico; ^14^ Center for Healthcare Policy and Research University of California Davis, Sacramento California USA; ^15^ St. Jude Children's Research Hospital Global Pediatric Medicine Memphis Tennessee USA

**Keywords:** adolescent and young adult, barriers, Caribbean, Central America, Latin America, oncology

## Abstract

**Background:**

Cancer is the fourth leading cause of death in adolescents and young adults (AYA) worldwide. Although successful treatment of cancer in AYA has increased in recent years in most of the world, this is not true for many low‐ and middle‐income countries (LMIC) where over 80% of all AYA live. This study investigated the needs of AYA with cancer in parts of Latin America (LATAM) through the perspectives of non‐physician health care providers and partners.

**Methods:**

Semi‐structured interviews (in Spanish) were conducted with non‐physician partners from Mexico, Peru, Central America, and the Caribbean over Zoom. Participants were recruited through previously identified local physicians and international non‐physician professionals working in these countries. Transcripts were coded and key themes identified until thematic saturation was reached (Atlas.ti).

**Findings:**

Thirty participants representing eight countries were interviewed, providing 1202 min of transcript data. Data were organized into barriers, facilitators, and strategies to improve the delivery of health care for AYA with cancer in LATAM at the patient‐ (e.g., financial barriers, continued schooling), parent‐ (e.g., limited medical literacy, advocacy), and hospital‐level (e.g., structural barriers, increasing funding).

**Interpretation:**

There are many similarities in the barriers and facilitators to AYA care between LATAM and high‐income countries (HIC); however, some characteristics are more unique to LATAM, for example, strict age restrictions for pediatric care and abandonment of therapy. As LATAM countries continue to build cancer control programs, there is an opportunity to consider our identified barriers, facilitators, and strategies to address the unique needs of AYA with cancer.

## 1 INTRODUCTION

Cancer is the fourth leading cause of death in adolescents and young adults (AYA: 15–39 years old) globally.^1^ Over 80% of AYA oncology patients worldwide reside in low‐and middle‐income countries (LMIC). Although mortality rates for AYA have recently decreased in most HIC due to innovations in treatment,^2^ this is not reflected across Latin America (LATAM).^3^ AYA are the primary workforce of these countries; therefore, the socioeconomic impact of improving care and outcomes for these patients could be substantial.^4^ Understanding the care of AYA with cancer in LATAM is crucial for identifying needs, guiding resource allocation, and to incorporate AYA‐specific plans into the development of cancer control programs.

The unique needs of AYA with cancer in high‐income countries (HIC) have been studied extensively.^5^, ^6^ The United States, Canada, the United Kingdom, Australia, and Italy have published strategies to improve AYA oncology care, such as access to clinical trials through organized networks, secondary prevention to minimize late effects of cancer therapies, and establishing a coalition of partners.^7^, ^8^ However, it is not clear that these strategies are applicable specifically in LATAM or other LMIC. In LMIC, studies have identified financial strain, transportation, and insurance status as major barriers to completing planned treatment for children and adolescents,^9^, ^10^, ^11^ but do not address the barriers faced by AYA. Therefore, we sought to identify the barriers and facilitators of AYA cancer care in LMIC, starting with LATAM, and potential strategies to improve this care is a critical first step.

## 2 METHODS

### 2.1 Participants

**FIGURE 1:**
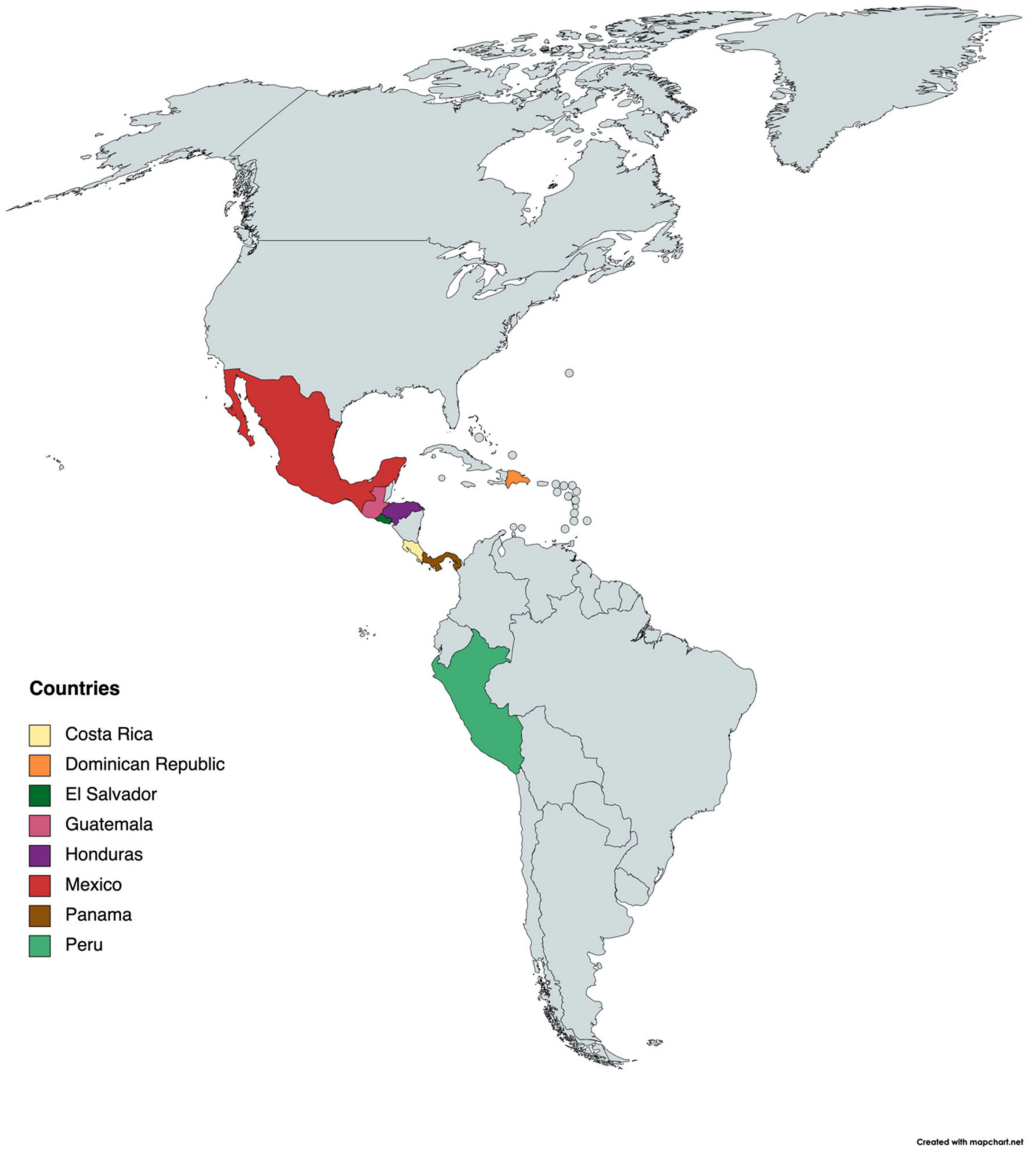
Map of the countries that participants are from.

We recruited a purposive sample including oncology nurses, social workers, nutritionists, psychologists, and non‐governmental organization (NGO) personnel who work with AYA with cancer from both pediatric and adult oncology settings across eight Latin American countries (Figure 1). Our group previously interviewed physicians.^12^ Participants were recruited by previously identified physician liaisons in each country and non‐physician partners. In addition, the recruited participants also provided additional contacts, therefore resulting in a snowball approach. The purpose of the study was read to all participants at the start of each interview and verbal consent was recorded prior to the start of the interview over zoom. The study was approved and deemed exempt by the University of California Davis Institutional Review Board prior to commencing the interviews.

### 2.2 Interview guide

The interview guide included demographic questions and open‐ended questions about: (1) General care of AYA patients with cancer, (2) Barriers to providing care for AYA patients and (3) Services and Strategies to improve AYA oncology care in participants' countries (Table S1).^13^ The interview guide was based on a previously administered survey and piloted with one of each of the following professionals in LATAM: nurse, social worker, psychologist, and NGO leader for clarity of questions. The interview guide was revised (EA and HN) based on the feedback received during the pilot interviews.

### 2.3 Participant interviews

From August 2021 to May 2022, interviews were conducted over Zoom in Spanish (CR). All interviews were recorded and transcribed verbatim with an anonymous ID number that only the research team had access to. Participants continued to be recruited until data saturation was achieved. Transcripts were de‐identified, transcribed in Spanish and translated to English by a professional translation service (GMR).

### 2.4 Data analysis

Utilizing Atlas.ti, a computer‐assisted qualitative data analysis software (ATLAS.ti, 2023), three researchers (EA, HN, SW) conducted a thematic analysis to identify themes across the interviews and organize them in major overarching themes. Researchers (SW and HN) employed open coding, an inductive coding technique that involves labeling concepts and defining categories based on the data, to develop an initial codebook.^14^, ^15^, ^16^ To ensure inter‐coder reliability, which refers to the consistency of coding between different researchers,^17^ SW and HN independently coded three interview transcripts and subsequently met to resolve any discrepancies. This step was crucial for enhancing the validity and reliability of the qualitative analysis. The refined codebook was then reviewed by EA and PF to ensure comprehensiveness and accuracy. Once the codebook was established through consensus, SW coded the remaining transcripts. Finally, codes were organized into categories, and categories were further consolidated into themes and subthemes by SW, HN, and EA. The code book is available to readers upon request.

## 3 RESULTS

### 3.1 Participants

Thirty participants from eight countries (six upper‐middle‐income and two lower‐middle‐income countries) in Latin America were interviewed, providing over 1202 h of transcript data (median length of interviews = 34 min) (Table 1). Participants were nurses (*n* = 11), psychologists (*n* = 8), NGO personnel (*n* = 5), social workers (*n* = 4), and nutritionists (*n* = 2). Participants had a mean of 14.6 years (standard deviation 8.47 years) of experience working in oncology. Participants in each category were drawn from multiple countries in LATAM. Fifteen participants who were solicited did not respond to multiple email invitations to participate.

**TABLE 1:** Characteristics of non‐physician adolescent and young adult professionals (AYA) from eight Latin American countries.

Characteristics	Total participants *N* = 30 *N* (%)
Profession	
Nurse	11 (36.7%)
Psychologist	8 (26.7%)
Nutritionist	2 (6.7%)
Social worker	4 (13.3%)
Other	5 (16.7%)
Sex	
Female	24 (80%)
Male	6 (20%)
Clinical practice	
Public	11 (36.7%)
Private	3 (10%)
Combination	6 (20%)
Other (NGO)	8 (26.7%)
Unknown	2 (6.7%)
Country of practice	
Costa Rica	3 (10%)
Dominican Republic	2 (6.7%)
El Salvador	2 (6.7%)
Guatemala	6 (20%)
Honduras	2 (6.7%)
Mexico	10 (33.3%)
Panama	2 (6.7%)
Peru	3 (10%)
Years of experience (mean, std dev)	14.6, 8.47

### 3.2 Themes

Non‐physician health care professionals highlighted major barriers and facilitators to caring for AYA with cancer in LATAM, as well as services they wished their health care systems had in place to benefit care for this population. Major themes were documented (Tables 2 and 3) and the frequency in which they were mentioned is in Table 4.

**TABLE 2:** Table of representative quotes of AYA specific themes in select Latin American countries.

Category	Theme	Example quotation
AYA specific barriers	Insurance	“A patient turns 18 years old and they lose their insurance, right? You have to treat them until they are 17 years old, with 11 months and 29 days. Yes, there have to be policies that favor the prolongation of coverage for vulnerable patients such as oncological patients.” (38) “So, even though I'm an employee and they discount from my check monthly for Social Security, if my daughter reaches 12 years of age, she's not covered anymore because my Social Security doesn't cover it, even though I am a contributor.” (16)
	Education	“The biggest complication they might face is missing several school days and then trying to go back after treatment because some of them miss entire school years and now they are in school with children who are much younger […]” (12)
	Need more AYA specific treatment sites	“So, having several sites or hospitals [for AYA care] would be great because the distance is also a barrier, right?” (10) “But a normal room in a hospital here has eight beds. They have communal bathrooms. There's a bathroom per floor.” (6) “[…] we're the only organization that sees children with cancer, and we only have one pediatric hospital nationally.” (18)
Facilitators for AYA care	Space designated for young people	“For example, we had a camp where we took the [AYAs] from Friday to Sunday, without their families, on their own. This is something that changed their lives and it made them realize that they could still do things.” (6) “We also do summer camps with the adolescents […]” (33)
	Supporting AYA survivors	“One of the things that I do is follow up after [AYAs] finished their treatment – to follow up with them once a year to find out what they're doing with their lives, what they're studying, what doubts they may have, how we can support them.” (18) “We've been organizing a gathering of 80 to 120 young adults every two years for 16 years to discuss these subjects like sexuality, education, participation, and volunteering.” (22)
	Existing AYA support groups	“Through this support group, adolescents like to be with other people their own age. They feel that they can identify with each other. They feel free to talk and express their fears and questions.” (3) “We also connect [AYA] patients who are going through the same experience.” (35)
	Providing resources	“I work a lot with psychological education. I explain to the patient what is happening and what are the expected emotions they will experience.” (15) “[…] the [AYA cancer] program covers the entirety of clinical care. It includes laboratory tests, imaging, and medical and surgical treatment […] for at least five years.” (37)
	Continuing Education	“The unit manages the [education] program of Let's Continue Learning in the Hospital, which allows them to continue [schooling].” (29) “The patient continues their educational activities online. There's a small school in [the shelter for families staying in the city for treatment]. There are workshops for moms.” (30)

**TABLE 3:** Table of representative quotes from categories and themes of cancer care in select Latin American countries.

Category	Themes	Example Quote (Participant ID)
Barriers	Financial barriers	“For example, I have young patients who have the financial power to buy a cellphone, a computer, pay for Internet connection, food, and other necessary things. They can even get medications that the public health system doesn't have […] There's a difference between those patients who have resources and the ones who don't, right?” **(**15) “I wish all [AYA] could have access to healthcare […] No child should remain without the right to obtain care for cancer because, cancer can be cured with these steps I've just mentioned.” (27)
	Language barriers	“We always try to have a translator of their language, but Guatemala has 23 or 24 languages. Sometimes, we do not have translators of all the Mayan languages.” (5) “So, depending on what part of the country they [AYAs] come from, sometimes we have to face this limitation, that they don't speak Spanish as their first language or that they don't know Spanish at all.” (7)
	Poor access to technology	“So, as you say, if we had to turn off the camera during a zoom session because of a connectivity issue then imagine what it's like outside the city where the signal is worse.” (12) “[…] it would be great to have an app that would remind patients when their appointment is, when they have to do a certain exam […]” (18)
	Traveling to receive treatment	“Another barrier, as well, is like, in my country […] the distances that they have to go to, to get to the hospital because many times they are long distances” (19) “They need to travel all the way to the capital to receive treatment.” (23)
	Religious barriers	“We have had patients who have a good socio‐economic standard of living and a good education but are very rooted in their religious beliefs and decide to abandon treatment.” (7) “Many of our families are prone to magical thinking, religious discourses. We validate those things but we [emphasize] that medicine is important.” (8)
	Conflicts between parents and providers	“And the part that I don't like sometimes is family interventions because sometimes they can be behavioral and they involve bad habits, lack of rules, complicated dynamics […]” (9) “[Parents] have a very strong will to overprotect their children.” (15)
	Limited medical literacy	“When they hear the word cancer it immediately translates to death. So, many patients take a long time to understand the illness's natural process and in understanding that there are many types of cancer and that not all of them lead to death, right?” (32) “For example, enteral nutrition and gastric tubes are very limited for these children because they believe it's more aggressive [treatment] for the patients.” (34)
	Caring for other children besides patient	“So, it's very difficult for them [AYA] to adhere to treatment because of the distance, because they [parents] have other small children at home [and] because they don't have anyone to help them with the children if they have to stay at the hospital for a long time […]” (10) “[…] both parents work, and they can't devote the necessary time [to come to the hospital] because they have several children.” (12)
	Difficulty in providing psychosocial support	“We also provide emotional support through our service of oncological psychologists, who are the psychologists who come here to support us. And we [the foundation] have to pay for their services.” (13) “The medical insurance plans don't cover psychological support. So, the patients have to pay it out of pocket.” (35)
	Continuing education for staff	“There aren't trainings for the staff. There aren't opportunities for internal or external graduate programs. There isn't any orientation in oncology… We earn very little money to be able to pay for a graduate program or keep studying.” (20) “I think that these constant training and education courses could also improve our teamwork abilities.” (23)
	Costs to hospital for treating patients with cancer	“The aspect that maybe isn't as pleasant is our limited ability to generate funds and the support that we receive from the state to achieve the foundation's necessary [inaudible].” (13) “[Treatments] cost thousands and thousands of dollars. I mean, it's practically inaccessible for most people.” (23)
	Structural barriers	“[…] we need more staff so we can provide better and more personalized care.” (10) “So, everything is like far away. So, if I have to go to the mental health department, we're on the 8th floor. Medical oncology is on the 8th floor. And if I want to go to the mental health ward, I have to walk for about 15 minutes.” (23)
	Limited treatment options	“We would love to have high‐quality medication. Even when we have very good medication, we have to go for a middle ground that is accessible for everyone.” (21) “[…] there are treatment methods and medications that haven't been updated in 20 years.” (34)
Facilitators	Educating patients and families	“In the case of adolescents, we orient the mother and the child about their self‐care and the family members about home care and alarming signs.” (25) “But I think that the way we offer them information is what makes the difference. Telling a child and telling a teenager are very different” (30)
	Multidisciplinary services	“We have a team called Integral Medicine and it's formed by the doctors in palliative care, social care, psychology, and child life, who are the psychologists who treat children and teenagers.” (10) “We have the psychology service, social workers, hemato‐oncology service, and nursing services too. We also have a school that had been closed during the pandemic but a month ago it was reopened.” (36)
	Comprehensive intake assessment	“Well, here in social work we provide care since the beginning […] We conduct an interview that allows us to learn more about their family context, their family situation, where they live, [and] their family dynamic. And based on that we determine the risk of treatment abandonment, right?” (8) “[…] when the patients first come here, my role is to see the parents, conduct a brief interview and collect information from the family and the [AYA] patient.” (10)
Strategies to Improve Care	Providing education for patients	“I think that there should be virtual platforms in our public health system where teenagers can access information about certain specific pathologies.” (15) “I have always fought about how they don't inform us about what is going on. Something that is lacking is education both [for the public] and professional.” (32)
	Providing more services for patients	“Another problem that patients have here is the lack of shelter when they come from distant places. The number of shelters [for AYA and a parent] that are available to them is very limited.” (6) “Evidently, no transportation, with the exception of some occasions in which [the foundation] supports them with half of the fare.” (29)
	Advocacy	“Well, I think that we could involve or raise awareness and sensitize people at schools, communities, right? About the importance of seeking treatment.” (9) “Yes, having campaigns where people visit communities to talk about childhood cancer and the types of childhood cancer that exist. That way people or families can catch it early on instead of realizing it when it's too late.” (10)
	Increase social support	“It's essential to be able to receive support from their family, as well as from their social network, the people who live around them, their church as well. When the person [AYA with cancer] doesn't belong to any such network, then, they may have all these complications during treatment.” (3) “So, what we want is for some relatives [of the AYA with cancer] to get to know each other and create a support network.” (37)
	Increasing funding and volunteers	“Although we receive public funds and donor funds, they do not give us enough to receive the entire [AYA] population.” (5) “Volunteers are what allow us to carry on with our work. The volunteers and I do everything.” (13)
	Need more medical resources within the hospital for providers	“[…] the financial resources are not enough to have a permanent translator here at the hospital.” (7) “Well, basically, for adolescents, we have patients with osteosarcomas. So, the majority of them required magnetic resonance and […] we don't have it in the unit.” (29)
	Need more staff and support for staff	“I think that we should have more staff to spend more time caring for the emotional side of patients. Sometimes we don't have enough time to talk or provide psychological attention.” (24) “Maybe, creating a support group for the staff who are dealing directly with these types of patients [AYA]. In personal experience, since I've been here so many years treating children and teenagers, there comes a time in which despite you not wanting it to, it affects you emotionally.” (29)

*Note*: Countries and roles of participants are not provided to maintain confidentiality.

**TABLE 4:** Table themes of cancer care in select Latin American countries and the frequency.

		Frequency	Types of participants
*AYA specific*			
Category	AYA specific barriers		
Themes	Insurance	10	Psychologist, nurse, other
	Education	17	Social worker, psychologist, nurse, other
	Need more AYA specific treatment sites	47	Social worker, psychologist, nurse, nutritionist, other
Category	Facilitators for AYA Care		
Themes	Space designated for young people	10	Social worker, psychologist, nurse, nutritionist, other
	Supporting AYA survivors	15	Social worker, psychologist, nurse, other
	Existing AYA support groups	20	Social worker, psychologist, nurse, other
	Providing resources	230	Social worker, psychologist, nurse, nutritionist, other
	Continuing Education	17	Social worker, psychologist, nurse, other
*General*			
Category	Barriers		
Themes	Financial barriers	34	Social worker, psychologist, nurse, nutritionist, other
	Language barriers	8	Social worker, psychologist
	Poor access to technology	5	Psychologist, other
	Traveling to receive treatment	30	Social worker, psychologist, nurse, nutritionist, other
	Religious barriers	11	Social worker, psychologist
	Conflicts between parents and providers	25	Social worker, psychologist, nurse, other
	Limited medical literacy	8	Psychologist, nurse, nutritionist, other
	Caring for other children besides patient	7	Social worker, psychologist, other
	Difficulty in providing psychosocial support	14	Psychologist, nurse, nutritionist, other
	Continuing education for staff	5	Social worker, psychologist, nurse
	Costs to hospital for treating patients with cancer	3	Social worker, nurse
	Structural barriers	80	Social worker, psychologist, nurse, nutritionist, other
	Limited treatment options	17	Psychologist, nurse, nutritionist, other
Category	Facilitators		
Themes	Educating patients and families	66	Social worker, psychologist, nurse, nutritionist, other
	Multidisciplinary services	57	Social worker, psychologist, nurse, nutritionist, other
	Comprehensive intake assessment	15	Social worker, psychologist, nurse, nutritionist
Category	Strategies to Improve Care		
Themes	Providing education for patients	12	Social worker, psychologist, nurse, other
	Providing more services for patients	26	Social worker, psychologist, nurse, nutritionist, other
	Advocacy	18	Social worker, psychologist, nurse, other
	Increase social support	9	Social worker, psychologist, nurse, other
	Increasing funding and volunteers	40	Social worker, psychologist, nurse, nutritionist, other
	Need more medical resources within the hospital for providers	44	Social worker, psychologist, nurse, nutritionist, other
	Need more staff and support for staff	32	Social worker, psychologist, nurse, nutritionist, other

#### 3.2.1 Themes Unique to AYA in LATAM

There were several themes that arose that were unique to the delivery of health care for AYA with cancer in LATAM at the patient‐, parent‐, and hospital‐level (Table 2).

### 3.3 Barriers specific to AYA

Barriers for patients included those specific to AYA in their stage of life in which they are coming into adulthood, studying in school or starting a career and perhaps a family. As one participant described,

“Young people must work, they want to be in the community, […] some already have partners, some have children. There are many reasons why sometimes these types of situations and barriers make it difficult for them to attend [treatment]. In the end, unfortunately they end up dying, they leave their treatment, or they come back to the hospital when they are sicker” (Participant 5).

Additionally, insurance is a particular barrier for many AYA. As one participant explained,

“Patients here in [Country], depending on the type of enrollment to health services they have are covered for different lengths of time […] So, these patients experience a watershed moment by the age of 16. This is the age when care is divided between pediatric and adult health services. […] They don't want to be referred to pediatric services and offering care for them in adult services is complicated. […] And patients with [insurance program] are in a complex situation because coverage, in theory, applies to patients who are 17 years old and 11 months. Public policies have not managed to extend total coverage for patients older than that. They are forced to switch to a hospital that provides care for adults [when they turn 18] and that no longer includes full coverage for their treatment. […] Also, in terms of coverage, the medical follow‐up services are not extended. That means […] if they relapse, or if they require specific procedures, they're not covered for any of those.” (Participant 22)

The difficulty in obtaining coverage for patients between childhood and adulthood was reported by other interviewees. They reported that this is how many patients become lost to follow‐up, as there are no exceptions to continuing coverage for patients with cancer once they reach a pre‐determined age specific to each country.

### 3.4 Facilitators for AYA care

Patient‐level facilitators for AYA care included existing support groups, having designated spaces for AYA, supporting AYA cancer survivors, providing psychological and financial support, and continuing patient schooling. Existing AYA support groups help create “a community of young people who all go through a similar situation” (Participant 6). AYA patients at some institutions receive psychological support and financial support with basic needs such as transportation, food, housing, and subsidized treatment. One NGO worker said,

“We give them money for transportation so they can travel from their place of origin all the way to the city. And once they are in the city, we provide a room for them [the AYA] and one parent or guardian […] We provide food. We provide transportation from the shelter to the hospital. We offer workshops and courses to empower parents a bit more” (Participant 12).

Furthermore, helping AYA continue their schooling while undergoing treatment was an important facilitator. One participant described how “[…] in the most intense part of the treatment where they spend a lot of time in the hospital […] there's a school inside the hospital” (Participant 17). Many of these facilitators already are in place at some participants' institutions. Supporting AYA cancer survivors was also important to participants, with one stating, “[…] we have to reinsert them into society through work, education, health, family and parental education” (Participant 8).

Lastly, increasing the number of AYA‐specific treatment‐centers was identified as important. While rare, some institutions have “a space that is specifically for young people” (Participant 13), like a camp for AYA or AYA‐specific sections of a ward. However, the majority of countries and institutions do not. One participant described the need for this: “Young girls or boys between the ages of 13 and 16 years old who are also experiencing a growth process require a more private environment […] These wards for teenagers don't exist” (Participant 22). Many participants pointed out that AYA often share facilities and physical spaces with children and adults:

“They could have a kid who was 14 or 15 years old next to an older adult of 80 or 85 years old […] The adult didn't want any noise […]. So, it was a space that was not adequate for a young person that age” (Participant 6).

#### 3.4.1 Themes general to cancer care in LATAM

Participants identified barriers, facilitators, and strategies to improve care that were more general to the environment of cancer care in LATAM, including pediatric cancer care (Table 3). These themes were divided into patient‐, parent‐, and hospital‐ levels.

### 3.5 Patient‐level barriers

Participants reported financial barriers, language barriers, poor access to technology, having to travel to receive treatment, and religious barriers. For example, one participant described, “Some families live 12 or 15 hours away from the capital. […] If they are not receiving any type of financial help to cover the transportation expenses, it affects them a lot” (Participant 8). Language barriers were described by several participants who reported that translators were not accessible at their institutions for non‐Spanish speakers. One said, “not being able to communicate with [patients] in their first language is difficult” (Participant 9). Many participants reported difficulties in helping patients and their families accept their diagnosis due to spiritual beliefs, while also acknowledging the important role of religion in forming a support network for patients and their families. One participant highlighted, “We need a professional who is an expert in religion so they can be a part of the team so we can offer spiritual support.” (Participant 15). Another said, “Sometimes, adherence to treatment will be influenced by the patients' beliefs. Sometimes, we need to seek their pastor for them to accept their situation” (Participant 3).

### 3.6 Parent‐level barriers

Reported parent‐level barriers included conflicts between parents and health care providers, limited medical literacy, and care needs of siblings. A participant described an example of the sometimes limited understanding of the seriousness of the situation at hand. “Another thing is that families […] understand that there is treatment, and that cancer is a serious illness, but they don't fully understand the seriousness of cancer, and many times they don't adhere to treatment” (Participant 10). The care needs of AYA patient siblings can make it difficult for parents to bring the AYA in for treatment. As one participant described, “Perhaps they have another seven children at home that can't be left alone. So, for example, in long treatments like leukemia that last two years, the family has […] to come [to the hospital multiple times] and not everyone can do so” (Participant 7).

### 3.7 Hospital‐level barriers

Hospital barriers for AYA care included difficulty providing psychosocial support, continuing staff education, hospital costs for treatment, structural barriers, and limited treatment options. Difficulty providing psychosocial support was related to costs of maintaining a psychologist on staff: “there isn't any durability in psychology staff, which is paid for by the foundation” (Participant 20). Continuing education for staff, including increasing knowledge about the care of AYA patients was also limited. A participant stated, “We earn very little money to be able to pay for a graduate program or keep studying” (Participant 20). The financial costs of treating patients with cancer for the hospital are also often high, as one participant described: “[…] there is a limited [hospital] budget and we don't have medication” (Participant 24). AYA who could afford to buy their medications out of pocket elsewhere were able to access chemotherapies that other AYA could not.

Structural barriers included the organizational and geographical separation of various clinics (i.e., the psychiatry department and oncology department may be in separate buildings), limited staff, and limited support services. Finally, limited treatment options can impact care. As one participant described, “We are not always able to provide the same protocol as in the United States due to the lack of medications” (Participant 7).

#### 3.7.1 Facilitators

Participants described several services and aspects of AYA care that they believed helped at the parent‐, or hospital‐level.

### 3.8 Parent‐level facilitators

Facilitators for parents included education about AYA cancer. For example, one participant described how they provide education to families after diagnosis. They said,

“We include all of the important points for the family about nutrition, how to do the consultation process [with the physician], the treatments they are going to receive, the protocol the patient is going to follow, the care in case of an emergency, what to do if they [AYA] have a fever […]. When they change treatment stages, we explain to them what we're going to do in that treatment stage” (Participant 17).

This participant further stated that this educational component helps reduce complications, especially in stages of treatment where AYA are particularly at high‐risk of infection.

### 3.9 Hospital‐level facilitators

The main facilitators for AYA care on a hospital‐level that participants noted were coordinated, multidisciplinary services, and a comprehensive intake AYA assessment. At one institution, a participant described how “all of the specialties participate [in the AYA care] like radiology, radiotherapy, the ones who work with nuclear medicine” (Participant 24). The comprehensive intake assessment was described as a facilitator for AYA care according to some participants. It was comprised of various disciplines including “child life, social workers, and hospice care for terminal patients” who work “in a trans‐disciplinary mode to […] identify protective [factors] and risk factors” for new patients (Participant 7).

#### 3.9.1 Strategies to improve care

Participants had strategies for how to improve AYA care in their home institution that would address patient‐, parent‐ and community‐, and hospital‐level barriers.

### 3.10 Patient‐level strategies

Suggested ways to improve AYA experiences include providing education for AYA on treatment, more support for survivors and more services in general for AYA. One wished that there could be an app such that a patient “…can see in the app what the risks or the events [side effects] that could come with [chemotherapy]. Or that he could see the symptoms that he could experience with the medication he's going to take” (Participant 30). Finally, many participants advocated for AYA specific services like housing, transportation, AYA support groups, “psychological services, [and] a social worker who can help people [AYA] who don't have the resources here in the capital,” (Participant 24) and a hospital education program to help AYA continue their schooling.

### 3.11 Parent‐level strategies

Two strategies were identified to improve care at the parent and family level. Increasing social support, such as allowing parents and friends to visit AYA while in hospital, would improve mental health for AYA patients while in hospital. Support groups for siblings of AYA were also suggested, because “when there's cancer in a family, the entire family needs help” (Participant 6).

### 3.12 Community‐level strategies

Participants emphasized the importance of advocacy for AYA cancer in the community. They wished for “campaigns that are directed towards the schools [to teach] the signs of early detection, which are the warning signs of childhood cancer,” (Participant 33). This may allow for an earlier diagnosis, which would lead to improved outcomes.

### 3.13 Hospital‐level strategies

Participants had ideas on how to improve care at the hospital level by increasing funding and volunteers, addressing the need for more medical resources within the hospital for providers, and considering the need for more staff and support for staff. For many participants, their institutions “…receive funding, but as a public institution, it's never enough” (Participant 7)—more government funding and help from volunteers, to support the patients during their treatment, is needed. Participants expressed the need for more machines for imaging (e.g., CT scanners) and medications, such as updating “the basic medication treatment [protocols] because they are very dated and that is also a limitation when we're trying to request a supplement” (Participant 34). Finally, many participants noted a need for more staff. One participant reported that “…in the hospital there are only 13 psychologists for a population of like 2,000 patients” (Participant 23).

## 4 DISCUSSION

This study assessed the barriers, facilitators, and strategies to improve cancer care delivery to AYA with cancer in six upper‐middle‐income and two lower‐middle‐income countries in LATAM from the perspectives of non‐physician partners. We identified specific themes that were unique to care for AYA with cancer in Latin America. Some challenges, such as financial barriers, limited psychological services, and the need to travel for treatment, are also seen in HIC, but were more prominent in these settings. Others are more unique to upper‐and lower‐middle‐income settings, such as abandonment of therapy, lower age cut‐offs for pediatric care and difficulty obtaining some medications.

AYA with cancer are a unique patient population. Challenges specific to this age group have been identified in our study in LATAM as a barrier to care and in HICs,^18^ specifically in the frustrations this patient population experiences, when facing the restrictions and limitations of their disease and treatment.^19^, ^20^ In addition, not having a distinct space for AYA patients to be together can exacerbate the isolation and disconnection they feel from their healthy peers. The need for an inclusive AYA space has been identified in similar studies of AYA patients with cancer in HIC.^21^ Strategies that have been implemented in one upper‐middle‐income country through the help of foundations, such as a separate hospital wards and recreational activities outside of the hospital for AYA, could be implemented in part of LATAM to help improve quality of life for AYA.

In addition, barriers to care observed in AYA with cancer included their often unique psychosocial needs. AYA patients with cancer can experience greater psychological distress; one study demonstrated that AYA with cancer have a higher prevalence of psychological distress compared to the general cancer population.^22^, ^23^ Many of our study participants described the important role that psychologists play in caring for AYA with cancer before, during, and after treatment. While lack of mental healthcare availability is not unique to LATAM, it may be exacerbated in these resource‐limited settings, where several participants described caring for patients with unstable housing, limited income, and food insecurity. The specific needs of these patients should be taken into consideration as cancer programs expand in these countries.

In identifying approaches to help improve care for AYA, there was strong support for multidisciplinary teamwork among participants in this study. Although some participants described an existing system where each patient is presented in a multidisciplinary team meeting, others expressed frustration at the lack of such a system. A report out of Australia demonstrated the importance of a multifaceted psychosocial support network, including documented care plans, multidisciplinary team meetings, and information about fertility preservation in the care of AYA with cancer.^24^ Implementing a multidisciplinary approach could have a substantial impact on the care delivered to AYA and in the inclusion of all participants who care for these patients.^25^

As with any study, this study has several limitations. The participants were personally recruited through known physician liaisons and international non‐physician professionals working with these countries. Thus, those interviewed are likely to be more motivated and engaged in improving care for the AYA population with cancer and their responses may not be generalizable. Translation of the original transcripts is also a limitation since some words in Spanish cannot be translated literally, and local expressions in one country may not be relevant in others. This study was conducted during the COVID‐19 pandemic, which may have impacted the availability of certain partners to participate as it impacted oncology care globally, and even more in resource‐constrained settings like LATAM. Finally, in some instances, it was difficult to distinguish the participant's perspective or opinion specifically for AYA from the common pediatric oncology challenges. However, the interview guide was AYA oncology focused and we were able to compare the experiences of AYA with cancer in LATAM to that of HIC. In addition, in order to maintain anonymity, we were unable to provide details on provider type by country linked with quotes. However, the number and variety of key participants recruited from multiple institutions and countries adds to the richness of the findings and is a strength. The addition of non‐physician staff from adult cancer centers together with childhood cancer centers allowed us to bridge the gap where many AYA are treated and improve inclusion for a more holistic perspective.

## 5 CONCLUSION

Given the breadth of specific needs of AYA with cancer, non‐physician partners have an important vantage point on their care experiences. This study highlights barriers and facilitators to care in LMIC in LATAM, which share both similarities and key differences with HIC. By focusing on the specific needs of AYA identified by professionals working most closely with them, resources can be allocated more efficiently and effectively to improve the care of this unique and often overlooked population with cancer. This study lays the foundation for collaborative work and advocacy at the regional and national level to improve outcomes for this unique patient population.

## AUTHOR CONTRIBUTIONS

**Samantha L. Wong:** Data curation (equal); formal analysis (equal); methodology (equal); writing – original draft (equal); writing – review and editing (equal). **Elysia Alvarez:** Conceptualization (lead); data curation (equal); formal analysis (equal); funding acquisition (lead); methodology (equal); resources (equal); supervision (equal); writing – review and editing (equal). **Emily E. Johnston:** Methodology (equal); supervision (equal); validation (equal); writing – review and editing (equal). **Crystal Romero:** Data curation (lead); formal analysis (equal); investigation (equal); writing – review and editing (equal). **Nuria Rossell:** Formal analysis (equal); methodology (equal). **Ligia Rios:** Data curation (equal); investigation (equal); writing – review and editing (equal). **Wendy Gómez García:** Conceptualization (equal); data curation (equal); resources (supporting); writing – review and editing (equal). **Federico Antillon‐Klussmann:** Data curation (supporting); investigation (supporting); resources (supporting); writing – review and editing (equal). **Ligia Fu:** Conceptualization (supporting); data curation (supporting); resources (supporting); writing – review and editing (supporting). **Soad Fuentes‐Alabi:** Conceptualization (supporting); data curation (supporting); resources (equal); writing – review and editing (supporting). **Karina Quintero Delgado:** Data curation (supporting); resources (supporting); writing – review and editing (supporting). **Daniel Ortiz Morales:** Data curation (supporting); resources (supporting); writing – review and editing (supporting). **Carolina Rodriguez‐Loza:** Investigation (supporting); resources (supporting); writing – review and editing (equal). **Salvador A. Lopez:** Data curation (equal); project administration (lead); writing – review and editing (supporting). **Melissa Gosdin:** Methodology (equal); supervision (equal); writing – review and editing (equal). **Marcio Malogolowkin:** Conceptualization (supporting); funding acquisition (supporting); writing – review and editing (equal). **Paola Friedrich:** Conceptualization (supporting); funding acquisition (supporting); methodology (supporting); resources (equal); supervision (lead); writing – review and editing (equal).

## FUNDING INFORMATION

Conquer Cancer Foundation Career Development Award, Daniel T. O'Connor, M.D. Memorial Research Grant.

## CONFLICT OF INTEREST STATEMENT

The authors have no conflict of interest to declare.

## PRIOR PRESENTATION

American Society of Pediatric Hematology Oncology, 2023.

## Supporting information


**Table S1:** Interview Guide for Key Partners (non‐physicians): Understanding the barriers and facilitators to providing and improving care for Adolescent and Young Adult patients.

## Data Availability

Datasets available from the authors upon request.
