# Supplementary material for: Perspectives of non‐physician partners on barriers and facilitators to AYA cancer care in Latin America
Source: Cancer Med. 2024 Oct 2;13(18):e70198. doi: 10.1002/cam4.70198 (PMC11447197; doi:10.1002/cam4.70198)
Supplement: Supplementary file 1 — Table S1: Interview Guide for Key Partners (non‐physicians): Understanding the barriers and facilitators to providing and improving care for Adolescent and Young Adult patients. [file CAM4-13-e70198-s001.docx]

**Supplemental Table 1:** Interview Guide for Key Partners (non-physicians): Understanding the barriers and facilitators to providing and improving care for Adolescent and Young Adult patients

*Begin Recording*

**Demographic Questions**

1. What is your profession?
2. What country are you from?
3. What country do you currently work in?
4. In what clinical setting do you work (if you work in a clinical setting)? Probe: private hospital, public hospital, oncology specific center?
5. How many years have you been working in your current profession?
6. How many years have you been working in your current role?
7. Do you work with adolescent and young adults with cancer?
   1. Do you specialize in taking care of patients in a particular age range? If so how old are the patients you take care of or work with?

**Interview Guide**

*General*

1. Tell me about your job.
   1. What is your role in take care of adolescents and young adults with cancer?
2. Tell me about your experience working adolescents and young adults? [Probe questions – What do you LIKE about caring for these patients. What do you NOT like? (etc.)]
   1. What is the minimum (if in adult setting) or maximum age (if pediatric setting) of patients you take care of or focus on (for foundations)?
      1. Would you feel comfortable taking care of patients younger/older than this age? Why or why not?
      2. What is the minimum/maximum age you feel comfortable taking care of? Why?

*Barriers to Providing Care for Adolescents and Young Adults*

1. Tell me about any barriers you face in caring for or working with adolescent and young adult patients with cancer.
   1. What services or strategies could decrease these barriers? Probe: Mental health services, financial aid etc
   2. What services or strategies do you think would be feasible to implement? Probe: mental health services, financial aid etc
   3. Which services or strategies to do you think are important but would not be feasible to implement?
2. Tell me about the barriers in your opinion that adolescents and young adults face when receiving treatment for their cancer?
   1. In your opinion are there any services or strategies that could decrease these barriers?
      1. What are they? Do you think they would be feasible to implement?
   2. Are there any services or strategies to do you think are important but would not be feasible to implement? Probe: Financial aid, fertility preservation services, etc

*Services and Strategies to Improve Adolescent and Young Adult Oncology Care*

1. Tell me about any services that you or your institutions provide for adolescents and young adults with cancer that you are think help them during their treatment.
   1. Are there any (of these services) that are specific to adolescents and young adults?
2. Tell me about what services you think would be helpful to have to improve care for adolescent and young adult patients with cancer.

*Conclusion*

1. Are there any other thoughts/concerns/experiences with regards to improving care for adolescent and young adults that you think are important for us to know about?
2. Are there any questions you think we should add to the interview?
